# The Luciferase Immunoprecipitation System (LIPS) Targeting the Spike Protein of SARS-CoV-2 Is More Accurate than Nucleoprotein-Based LIPS and ELISAs for Mink Serology

**DOI:** 10.1155/2023/1318901

**Published:** 2023-02-22

**Authors:** Agathe Auer, Alessio Bortolami, Francisco J. Berguido, Francesco Bonfante, Calogero Terregino, Alda Natale, Alice Fincato, Barbara Colitti, Sergio Rosati, Charles E. Lamien, Giovanni Cattoli

**Affiliations:** ^1^Animal Production and Health Laboratory, Joint FAO and IAEA Centre for Nuclear Applications in Food and Agriculture, Department of Nuclear Sciences and Applications, International Atomic Energy Agency, Friedenstrasse 1, A-2444, Seibersdorf, Austria; ^2^Emergency Prevention System (EMPRES), Animal Health Service Food and Agriculture Organization of the United Nations (FAO-UN), Rome, Italy; ^3^Istituto Zooprofilattico Sperimentale Delle Venezie (IZSVe), Padua, Italy; ^4^Department of Veterinary Sciences, University of Turin, Turin, Italy

## Abstract

Since anthropo-zoonotic outbreaks of SARS-CoV-2 have been reported in mink farms, it is important to monitor the seroprevalence within this population. To investigate the accuracy of nucleo (*N*) or spike (*S*) protein-based assays to detect anti-SARS-CoV-2 antibodies in animal serum, we compared four assays, two commercial *N*-based enzyme-linked immunosorbent assays (ELISA) validated for animal sera and two luciferase immunoprecipitation systems (LIPS-N and LIPS-S), to the reference standard plaque reduction neutralisation test (PRNT). Samples included in this study were derived from a naturally infected mink population. For the first time in this study, serum samples of mink were collected over a 307-day period, at different time points, thus providing an overview of performances of four different rapid serological tests over time. The assays were compared by performing a correlation analysis using *R*2, Spearman's rank-order correlation coefficient, and Fleiss' and Cohen's kappa for analysis of agreement to PRNT, and an UpSet chart was created to visualize the number of shared positive samples between assays. Cohen's kappa test on categorical data showed an excellent agreement between PRNT and LIPS-S, while agreements between PRNT and *N*-based methods decreased from fair for LIPS-N to poor agreements for the ELISA kits. In addition, LIPS-S revealed the highest number of true-positive SARS-CoV-2 samples compared to *N*-based methods. Despite an excellent agreement between LIPS-S and PRNT, a weak correlation was detectable between PRNT titres and relative light units. This study shows that the LIPS-S assay can be used for serological surveillance within a naturally exposed mink population, while *N*-based serological assays are less accurate providing a higher number of false-negative results, especially at a later stage of infection, thus indicating that *N* antibodies are less persistent in naturally exposed mink. Our findings provide crucial information for veterinarians and competent authorities involved in surveillance and outbreak investigation in wild and farmed minks.

## 1. Introduction

Despite being fast, reliable, and highly sensitive, molecular methods only detect viral RNA from active infections [1]. Therefore, serological testing is preferable in some situations, including tracking virus circulation in species that may not be susceptible to clinical disease, outbreak investigation, and identifying new susceptible hosts [2–6].

Serological methods detecting antibodies against the severe acute respiratory syndrome coronavirus 2 (SARS-CoV-2) include indirect immunofluorescence assay (iIFA), enzyme-linked immunosorbent assay (ELISA), chemiluminescence immunoassays, lateral flow immunochromatographic assay, luciferase immunoprecipitation system (LIPS), and neutralization-based methods, such as virus neutralisation test (VNT) or plaque reduction neutralisation test (PRNT) and surrogate VNT (sVNT) [7, 8]. While PRNT is a commonly used gold standard for assessing immunity against coronavirus infections, it is time-consuming and requires high biosecurity laboratories and personnel trained to handle live SARS-CoV-2; therefore, only a few laboratories can perform this type of analysis [9, 10]. Furthermore, for population-based seroprevalence studies, where the sample size is usually large, PRNTs are impractical. Therefore, high-throughput assays such as ELISA, LIPS, and sVNTs are advantageous, as they do not use live viruses and do not require Biosecurity Level 3 (BSL3) facilities. Commercial sVNTs for animals, e.g., the cPass™ Technology sVNT kit from GenScript, are available and have been tested on mink. However, a recent study has found that sVNT lacks sensitivity at low titres, and results should only be interpreted qualitatively or semiquantitatively rather than quantitatively [11]. In addition, sVNT is more laborious than ELISA and LIPS.

The genome of SARS-CoV-2 encodes four principal structural proteins: spike (*S*), nucleoprotein (*N*), membrane (*M*), and envelope (*E*). *S* protein mediates attachment to host cells and virion entry and contains the receptor-binding domain (RBD) [12]. The full *N* and *S* proteins and different *S* domains, including RBD, have been used as antigens for testing the SARS-CoV-2 humoral immune response [13]. In a previous report, we evaluated the use of LIPS assays targeting the *S* and *N* protein (LIPS-S and LIPS-N) on five different animal species, including mink [7]. We showed that while the LIPS-S assay produced good discrimination between the positive and negative samples with 100% agreement with PRNT, the LIPS-N assay did so less accurately. Furthermore, a recent study demonstrated that a correlation between VNT and N-targeted serological assays is mediated by the concomitant presence of RBD or *S*1/*S*2 proteins on the *N* protein [14]. However, currently, the only commercially available serological assays for animals target the N protein [15–18].

As a result of the high susceptibility of minks to SARS-CoV-2 infection, outbreaks have been reported in numerous mink farms in Europe, the United States, and Canada [1, 15, 16, 19]. Due to mutations on the *S* protein of SARS-CoV-2 in minks, spillback events from minks to humans have been reported, leading to the culling of mink in Denmark [20]. According to EFSA [2], SARS-CoV-2 infection in minks needs to be monitored and investigated to understand the impact of mutations occurring in this species on the viral fitness, contagiousness, pathogenicity, immunotherapy, and vaccine efficacy in human populations [19]. Furthermore, “for monitoring purposes, it is important to know the time period from and until when SARS-CoV-2 infection can be detected” [2]. Therefore, high-throughput and validated serological assays for SARS-CoV-2 are of extreme importance for the accurate monitoring of SARS-CoV-2 circulation in wild and farmed minks. As *N*-based ELISA kits are available on the market, it is important to collect information on their performances under field conditions to ensure quality of results and proper data interpretation. Here we compared five assays, the PRNT, the LIPS-S, and the LIPS-N assay, and the two commercially available ELISAs currently marketed for animal species, ID Screen® SARS-CoV-2 Double Antigen Multi-Species ELISA (IDV) and ERADIKIT™ COVID19-MULTISPECIES (Eradikit), both targeting the *N* protein. For these ELISAs, we lack data on their performances, including the level of agreement between the assays and the gold standard PRNT, for sera derived from naturally exposed mink farms. Furthermore, for the first time, in this study, we compared the different serological tests for their ability to detect antibodies against SARS-CoV-2 in a mink population over a long period of time (307-day).

## 2. Materials and Methods

Table 1 displays sampling details, and Table 2 lists the assays used in this study. The first sampling trip indicated as Day 0 refers to the official notification date to WOAH.

### 2.1. Sample Description

Samples were collected in the framework of a national surveillance plan from a single Italian mink farm for pelt production, housing 3379 minks (Neovison vison). The farm was declared infected on the 18th of March 2021 and reported to the OIE (https://wahis.oie.int/#/report-info?reportId=32262) when one out of sixty oropharyngeal swabs collected at the same time tested positive for SARS-CoV-2 by RT-qPCR and all the sera collected were found positive by PRNT. The farm was then monitored every two weeks by RT-qPCR according to the protocol described in [22], targeting fragments of the E, *N,* and RdRP genes on RNA extracted from oropharyngeal and anal swabs collected from 30 randomly selected animals. Apart from the one swab sample that tested positive on the first day of collection (sampling reference *A*), the virus was never detected by RT-qPCR. This indicates that the animals were not exposed to recurrent waves of SARS-CoV-2, and the serological results relate to either convalescent or fully recovered animals after the end of an outbreak. No symptoms or abnormal mortality was observed in the mink population at any time during the period under investigation. On four separate occasions, sampling references *A*–*D* (Table 1), blood samples were taken from the cranial vena cava of 30 randomly selected animals (different animals each time) to monitor the presence of antibodies against SARS-CoV-2 as described in Table 2. Due to difficulties in obtaining a large amount of serum from each animal, only samples that allowed sufficient blood volume to perform multiple serological tests were included in the present study (Table 1). Sample classification as true positive or true negative was based on PRNT.

### 2.2. Preparation of PRNT, ELISAs, and LIPS Assays

#### 2.2.1. Plaque Reduction Neutralisation Test (PRNT)

The negative or positive status of each serum was confirmed by PRNT, as previously described in [21]. In brief, after heat inactivation, samples were diluted in Dulbecco's Modified Eagle Medium (DMEM) and then mixed with a virus solution containing 20–25 focus-forming units (FFUs) of SARS-CoV-2. A parental SARS-CoV-2 strain (hCoV-19/Italy/PD_20VIR1935i-P4-L/2020-B.1 lineage) isolated during the first wave of the pandemic in March 2020 was used for the assays. Since during the sampling period, December 2020 to January 2021, the dominant variant was B.1 in Italy with only a modest circulation of the antigenically similar English variant (Alpha or B.1.1.7), we assume that the virus used to perform the PRNT has a high degree of antigenic identity with the viruses that infected the minks. After 1 h at 37°C, 50 *μ*L of the virus-serum mixtures were added to confluent monolayers of Vero *E*6 cells in 96-well plates and incubated for 1 h at 37°C in a 5% CO_2_ incubator. After 26 h of incubation and cell fixing, visualization of plaques was performed with an immunocytochemical staining method using an anti-nucleoprotein monoclonal antibody (1 : 10,000; Sinobiological) for 1 h, followed by 1 h incubation with peroxidase-labelled goat anti-mouse antibodies (1 : 1,000; DAKO) and a 7 min incubation with the True Blue™ (KPL) peroxidase substrate. Foci were visualized on BioSpot™ (CTL Europe GmbH). The serum neutralization titre was defined as the reciprocal of the highest dilution, resulting in a reduction of the control plaque count >50% (PRNT_50_). Tests were run in duplicate.

#### 2.2.2. Enzyme-Linked Immunosorbent Assays (ELISAs)

Serum samples were tested to evaluate the presence of IgG anti-SARS-CoV-2 nucleocapsid antibodies using the ID Screen SARS-CoV-2 Double Antigen Multi-Species ELISA Kit and the Eradikit COVID19-MULTISPECIES according to the manufacturer's instructions. The ERADIKIT™ In3Diagnostic, COVID19-MULTISPECIES ELISA kit is a double-antigen ELISA test to identify immunoglobulins, including IgM, IgG, and IgA, against SARS-CoV-2 in animal serum used in the framework of a national surveillance plan to investigate SARS-CoV-2 in domestic animal species in Italy.

#### 2.2.3. Luciferase Immunoprecipitation System (LIPS)

The pREN2 and pGaus3 plasmids encoding genes for the luciferase fusion proteins of SARS-CoV-2 *N* and *S* proteins were utilized [23]. The assays were performed as previously described in [7]. In brief, Cos 7 cells grown in DMEM supplemented with 10% foetal bovine serum were transfected using the FuGENE 6 protocol (Promega) with plasmid pREN2 SARS-CoV-2 Nucleocapsid for the SARS-CoV-2-LIPS-*N* assay or pGaus3 SARS-CoV-2-Spike for the SARS-CoV-2-LIPS-S. Two days after transfection, the medium was removed and the cells were washed with 6 ml of phosphate buffered saline (PBS), followed by the addition of 1.4 ml of cold lysis buffer ((50 mM Tris, pH 7.5, 100 mM NaCl, 5 mM MgCl_2_, 1% Triton *X*-100, 50% glycerol, and protease inhibitor (2 tablets of complete protease inhibitor cocktail (Roche) per 50 ml of lysis buffer)). Cells were then harvested and dissociated using a sonicator (Vibra-Cell VCX 750, Sonics and Materials Inc., Newtown, CT, USA). The samples were then centrifuged at 16,000 g for 4 min at 4°C, and the supernatants were collected and stored at −80°C until required. To determine total luciferase activity, 1 *μ*l of crude fusion protein extract was added to 100 *μ*l of coelenterazine substrate (Promega) in a white 96-well plate (Sterilin). Relative light units (RLU) were measured with a luminometer (Berthold Centro LB, Berthold Technologies, Bad Wildbad, Germany) for 5 s, and the volume of protein extract required to produce 1 × 107 RLU was determined.

LIPS assays for measuring antibodies in the serum samples were carried out by mixing 40 *μ*l of buffer *A* (50 mM Tris, pH 7.5, 100 mM MgCl_2_, 1% Triton *X*-100), 10 *μ*l of diluted serum (1 in 10 in buffer *A*), and 50 *μ*l of buffer A containing enough fusion protein extract to generate 1 × 107 RLU (as calculated above) in each well of a 96-well plate. This mixture was incubated for 1 h at room temperature with gentle shaking. The mixture was then transferred to a 96-well Multi-Screen HTS filter plate (Millipore) and incubated with 5 *μ*l of Ultralink immobilized protein A/G beads (Pierce Biotechnology Inc.) for 1 h at room temperature with gentle shaking and then washed 8 times with buffer *A* and twice with PBS using a vacuum manifold. Coelenterazine substrate was added, and the light emission was read for 5 s using a luminometer (Berthold Centro LB, Berthold Technologies, Bad Wildbad Germany). Samples were tested in duplicate on two separate days for a total of four replicates.

### 2.3. Correlation and Interassay Agreement

Two approaches were used to compare the various diagnostic assays: qualitative analysis of methods and quantitative analysis of methods.

#### 2.3.1. Qualitative Analysis of Methods

Fleiss' kappa test was performed on categorical data (positive or negative) to assess the reliability of agreement between all the methods using the Interrater Reliability (IRR) package in *R*. For the pairwise agreement analysis, Cohen's kappa tests were performed using the Visualizing Categorical Data (VCD) package in *R*. The mean RLU values from two independent runs were used. Statistical analysis and data representation were carried out using the *R* statistical package. Box plots were used to visualize the samples per group of the positive and negative population for LIPS assays. For the visualization of shared positive samples between the assays, an UpSet plot [24] was created using the package “UpSetR” [25].

#### 2.3.2. Quantitative Analysis of Methods

A quantitative agreement between LIPS-S and PRNT was visualized using the ggplot2 package and by performing a correlation analysis using Spearman's rank-order correlation coefficient.

## 3. Results

### 3.1. Qualitative Analysis of Methods

**Figure 1:**
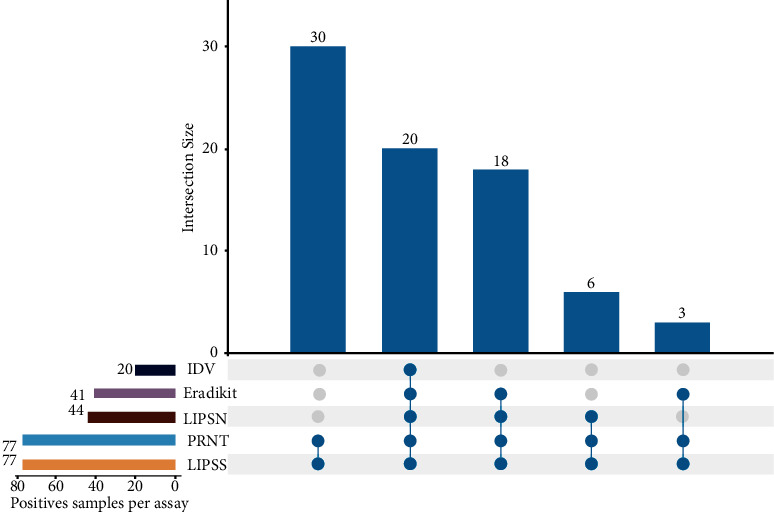
UpSet plot showing relationships of PRNT, LIPS-S, LIPS-N, Eradikit, and IDV. UpSet is sorted according to shared positive results between the assays (*n* = 77).

Figure 1 shows the relationship between assays based on shared positive results. For example, the LIPS-S and the PRNT shared 77 positive results, the LIPS-N and PRNT/LIPS-S shared 44, Eradikit and PRNT/LIPS-S share 41, and IDV has 20 positive results in common with PRNT/LIPS-S. On the other hand, 30 positive samples were detected only by PRNT and LIPS-S.

Fleiss' kappa value was 0.32, indicating a fair agreement between all assays.

**Figure 2:**
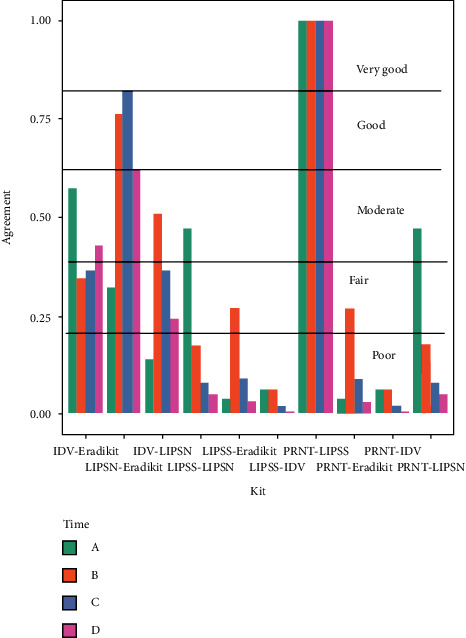
Cohen's kappa agreements for each time point (A–D) between IDV and Eradikit, LIPS-N and Eradikit, LIPS-N and IDV, LIPS-S and LIPS-N, LIPS-S and IDV, PRNT and LIPS-S, PRNT and Eradikit, PRNT and IDV, and PRNT and LIPS-N.

The pairwise agreements determined by Cohen's kappa test are presented in Table 3. The variations in the strength of agreement between assays at each sampling point (*A*–*D*) are shown in Figure 2. Cohen's kappa agreement is interpreted as poor for values below 0.20, fair for values between 0.2 and 0.4, moderate for values between 0.4 and 0.6, good for values between 0.6 and 0.8, and very good for values above 0.8. Cohen's kappa test on categorical data (positive or negative) showed excellent strength of agreement between the PRNT and LIPS-S (Table 3) at all sampling points (Figure 2). The agreement between PRNT and LIPS-S and other methods decreased from fair for LIPS-N (0.2845) to poor for Eradikit (0.162) and IDV (0.08501) (Table 3). The agreement between LIPS-N and either LIPS-S or PRNT was moderate at sampling point A and poor at all other sampling points. The agreement between Eradikit and either LIPS-S or PRNT was poor at time points *A*, *C*, and *D* and fair at time point *B* (Figure 2). The agreement between IDV and either LIPS-S or PRNT was poor at all sampling points (Figure 2).

The Eradikit and IDV kits showed an overall moderate agreement (0.414) in detecting positive mink samples (Table 3); the agreement was moderate for time points *A* and *D* and fair at time points *B* and *C* (Figure 2). LIPS-N had an overall moderate agreement with IDV (0.4267) and a good agreement with Eradikit (0.56) (Table 3). Agreements between LIPS-N and IDV were poor at time point *A*, moderate at time point *B*, and fair at time points *C* and *D*. The strength of agreement between LIPS-N and Eradikit was fair at time point *A* but was good at all other time points (Figure 2).

**Figure 3:**
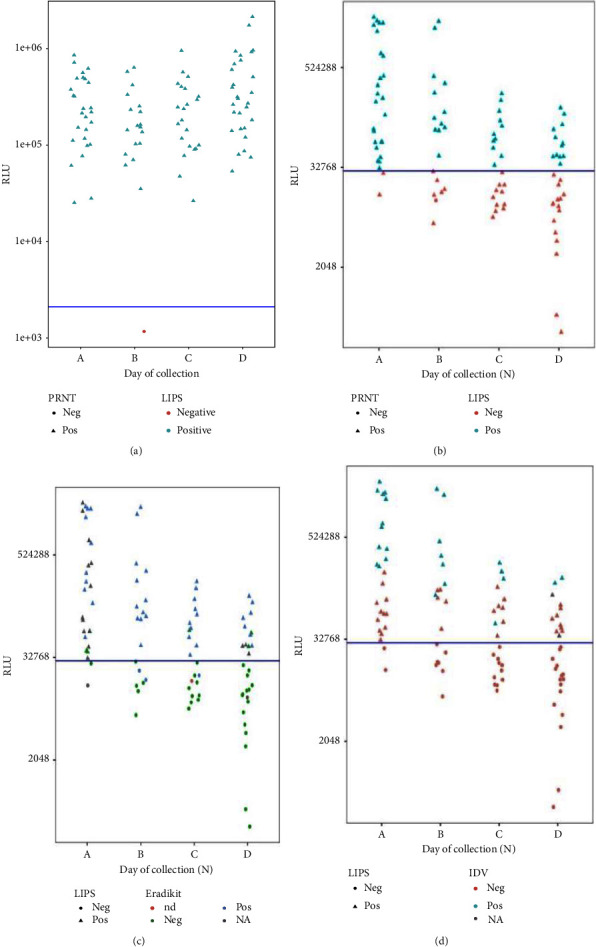
Distribution of RLU values generated using the sera from naturally SARS-CoV-2-infected mink samples across (a) the threshold line for the LIPS-S assay with the indication of PRNT results and sera sampling dates, (b) LIPS-N assay with threshold line, identification of PRNT results, and sera sampling dates, (c) LIPS-N assay with threshold line, identification of Eradikit results, and sera sampling dates, and (d) LIPS-N assay with threshold line, identification of IDV results, and sera sampling dates. Negative samples are located below the blue threshold line of mean plus five standard deviations.

For further comparative analysis of the LIPS assays and also to compare *N*-based assays against each other, all samples were plotted as RLU per sampling date (*A*–*D*), with the results (positive/negative) from other methods indicated in Figure 3. The results show that the LIPS-S assay correctly identified all samples according to their positive and negative status independent of the serum collection time determined by the antibody neutralization test (Figure 3(a)). Unlike LIPS-S, the RLU values generated by LIPS-N from PRNT positive sera declined over time, and an increased number of PRNT positive sera fell below the LIPS-N threshold line (Figure 3(b)).  Figure 3(c) shows that RLU values generated from naturally infected mink decreased over time using LIPS-N and shows false-negative results and undetectable samples obtained from Eradikit.  Figure 3(d) shows RLU values from the same sample set over time using LIPS-N, with false-negative results obtained from IDV remaining the same.

**Figure 4:**
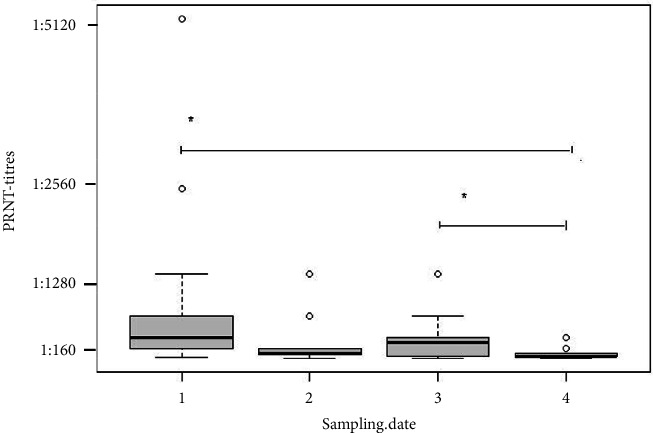
Boxplot of PRNT titres at each sampling point using pairwise comparison with the Wilcoxon rank sum test and Bonferroni adjustment (alpha = 0.05).

In Figure 4, PRNT titres from each sampling date are plotted. Pairwise comparisons using the Wilcoxon rank sum test with Bonferroni adjustment showed that PRNT titres from the first and third sampling dates are significantly higher than those of the last sampling date (Figure 4) (*p* < 0.05).

There was a significant difference in RLU values (pairwise *t*-test, *p* < 0.05) between true-positive (*n* = 77) and true-negative samples (*n* = 12) as measured by PRNT for both LIPS-S and LIPS-N assays. The mean RLU values for LIPS-S and LIPS-N based on positive samples were 281516 and 233766, respectively; the mean RLU values for LIPS-S and LIPS-N obtained from negative samples were -1865 and 8855, respectively.

### 3.2. Quantitative Analysis of Methods

**Figure 5:**
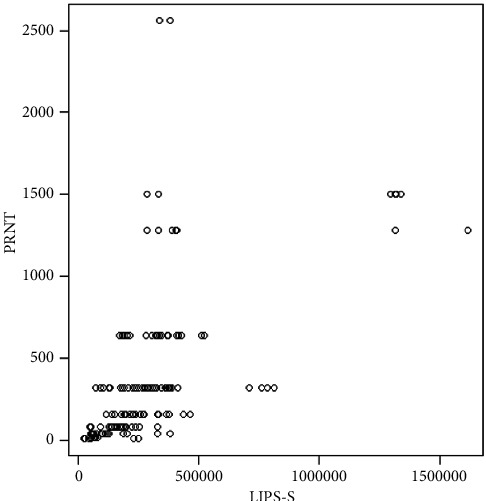
Visualization of the correlation between LIPS-S and PRNT using Spearman's rank-order correlation coefficient.

Since both ELISAs have already been validated by their respective manufacturer and do not provide a correlation between *S*/*P* values and PRNT titres, we investigated the correlation between RLU values from LIPS-S and PRNT titres. There was no significant difference between RLU values obtained from high (≥1 : 640) and low (<1 : 640) titres with a weak *R*2 value of 0.3262579. In Figure 5, RLU values from LIPS-S are plotted against titres measured by PRNT. Spearman's rank-order correlation coefficient between LIPS-S assays and PRNT was weak with 0.3782656.

## 4. Discussion

WOAH has defined COVID19 as an emerging disease in animals and is promoting the implementation of surveys on the prevalence of infection in animals [26]. Mink farms represent a serious, unrecognised animal reservoir for SARS-CoV-2 [20], and since the start of SARS-CoV-2 pandemic, multiple countries have reported outbreaks in farmed minks [19], with serious implications for public health due to the risk of viral mutation and spillback events [1]. Therefore, the availability of laboratory tools for molecular and serological surveillance with well-defined characteristics is essential for proper application and result interpretation.

Compared to the assays based on the *N*-antigen tested in this study, the LIPS-S assay is more accurate and comparable to the gold standard PRNT, detecting the greatest number of true-positive mink samples (Figures 1–3(a)). This finding is in accordance with other published data on animals and confirms the main finding in our previous report using LIPS assays targeting the *S* and *N* protein [7]. However, in this study, we also show inferior sensitivity and a progressive loss of performance at later time points of infection from ELISA and LIPS assays targeting the *N* protein, indicating that the *S* protein is a more reliable target than the *N* protein for serological assays applied to sero-surveillance activities in mink populations. Furthermore, this study demonstrates that LIPS-N gives fewer false-negative results than the selected ELISAs targeting the *N* protein.

To date, there is no commercial ELISA kit available targeting the *S* protein which is validated for use in animals. However, several in-house ELISA assays targeting the *S* protein have been developed and deemed fit for serological screening in animals. A recent study reported that antibody titres against the *N* protein in sera of vaccinated cats and dogs were too low to be detected using the IDV assay [18]. In contrast, their in-house indirect ELISA, targeting both *S* and *N* proteins, detected SARS-CoV-2 antibodies in vaccinated animals. They concluded that the N protein concentrations in vaccinated cats and dogs were much lower than *S* protein concentrations and confirmed their findings by immunoblotting. The study in [27] reported similar findings by developing ELISAs targeting *N* and *S* proteins, respectively, and comparing these assays to VNT on canine and feline sera. The authors reported a poor correlation between VNT and the ELISA targeting the *N* protein and recommended excluding the N protein as an antigen for serological screenings of cats and dogs. Pulido et al. developed two ELISAs and a duplex protein microarray immunoassay to detect SARS-CoV-2 antibodies specific to the *S* protein's receptor-binding domain (RBD) and to the *N* protein, respectively. They found that the RBD was a more sensitive target for surveillance in mink sera, although they did not compare their assays to VNT. In contrast, a recent study in humans showed that LIPS using antibodies against the *N* protein is more sensitive than the detection of antibodies against the *S* protein and that *N* antibodies generally appear earlier than spike antibodies [23]. Sera analysed in [23] derived from patients with positive PCR results only indicated an early stage of infection, whereas samples from animals analysed by other authors and the ones included in this study were derived from a later stage of infection [13, 18, 27]. Different results obtained in [23] on humans compared to published data on animals might be due to different sera origins or that anti-*N* antibodies appear earlier but also decrease faster than anti-*S* antibodies.

That anti-*N* antibodies decrease faster than anti-*S* antibodies is supported by other more recent publications on animals and humans [17, 28]. The study in [28] developed an experimental double antigen-based ELISA targeting the *N* protein for humans and pet animals including cats and dogs and compared their results to PRNT. They found false-negative results tested by ELISA with sera at PRNT titres below 1 : 640 and concluded that differences may be explained by different kinetics in antibodies raised against *N* and *S* protein and suggest that the use of the *N* as antigen in SARS-CoV-2 infections is of importance in an early stage of infection [28]. The study in [17] compared sera from infected cats and dogs using VNT, PRNT, IDV, and Eradikit and showed that neutralizing antibodies persist up to 10 months, whereas IDV and Eradikit showed false-negative results throughout their study period. The study in [17] suggested that cats and dogs may develop a long-term neutralizing antibody response against SARS-CoV-2. Our results lead to similar conclusions in mink. Thus, when the time of infection is not known, it is suggested to use assays targeting the *S* protein or both (*S* and *N* protein) or to confirm results using VNTs. One long-term study in mustelids could find anti-SARS-CoV-2 antibodies using an in-house ELISA targeting the *S* protein in pet ferrets. In this study, of 127 animals, only two were seropositive for SARS-CoV-2 of which one resulted as seronegative in a serum sample obtained 128 days after the ferret had tested positive for the first time. The other animal remained seropositive in a sample taken 129 days after the first positive result. However, they did not compare their results to *N*-based methods nor tests measuring neutralizing antibodies, and the experiment was terminated after 129 days [29].

Despite an excellent qualitative agreement between PRNT and LIPS-S, only weak Spearman's rank-order correlation coefficient between RLU values and PRNT titres was measurable with no significant differences between RLU values obtained from high (≥1 : 640) and low (<1 : 640) titres. In a previously published paper by [7], Spearman's correlation coefficients were decreasing over time and in this study, samples from even later time points of infection were analysed. The differences in sampling times might have influenced the correlation between RLU values and PRNT titres in this study. Further analysis is required to evaluate LIPS as a quantitative assay, e.g., using serial dilutions of quantified sera from one animal and applying VNTs and LIPS simultaneously in triplicate.

The N-based methods detected a lower number of true-positive samples; LIPS-N was the most accurate in detecting true-positive samples of the three methods, followed by Eradikit and then IDV (Figure 1). Since both ELISAs target multiple species, including mink, ferret, feline, canine, bovine, ovine, caprine, and equine, a loss of sensitivity is to be expected. SARS-CoV-2 is a notifiable disease, and according to EFSA, it is recommended to monitor SARS-CoV-2 infection in mink [2, 6, 26]. It is therefore particularly important to have a sensitive test at hand when testing mink for SARS-CoV-2, which will help tracking the circulation of this virus and prevent spillover events. A limitation of this study is that we do not know whether samples at each time point were from the same animal; therefore, the natural kinetic of the neutralizing antibodies detected by the PRNT assay for individual animals could not be established. Nevertheless, on the farm level, the PRNT titres remained positive, although decreasing over time. Pairwise comparisons using the Wilcoxon rank sum test with Bonferroni adjustment showed that PRNT titres from the first and third sampling dates are significantly higher than from the last sampling date (Figure 4). That neutralizing antibodies in animals over a time period of 13 months wane but persist has been shown in [30] and match our findings. It is interesting to note that more negative results in the last sample collection time point (*D*) were consistently observed in all *N*-based assays (Figure 3). This finding suggests that *N* antibodies are less abundant and less persistent in naturally exposed mink. This would support the previous study on vaccinated cats and dogs [18] and in naturally exposed companion animals [31] and similar observations in humans where a faster waning of anti-*N* antibodies levels compared to anti-RBD antibodies has been reported [32, 33].

The study lacked mink sera positive for other coronaviruses and pathogens, and a limited number of true-negative mink sera were included, but the analytical specificity could not be calculated.

In conclusion, these experiments demonstrate that the *S* protein is a more accurate target than the *N* protein for serological assays aiming to detect past infection in mink populations, with excellent agreement with a gold standard method. Furthermore, when applying assays aimed at the detection of anti-*N* antibodies, our results suggest that the time of infection and the choice of assay, LIPS or ELISA, in addition to the sampling size, should be taken into consideration to reduce false-negative results. This is crucial information for the selection of tests to be used in serological investigations in animal populations, as serosurveys can provide valuable information about past infections, silent virus circulation, and outbreak evolution. Our data will provide useful evidence for the design of postvaccination sero-monitoring and surveillance in mink farms where vaccination against SARS-CoV-2 is applied (“ODA requires all captive mink vaccinated against SARS-CoV-2, the animal virus linked to COVID19 in humans,” 2021; [34]. For minks vaccinated with vaccines eliciting a response solely against the spike protein, postvaccination surveillance to monitor the circulation of the virus can rely on anti-*N* serological assays if properly timed. Nonetheless, we recommend cautious use of anti-*N* serological assays as DIVA tools, as different assays might have significantly different sensitivity and temporal windows within which the identification of *N*-positive animals is accurate. Due to the continuous evolution of SARS-CoV-2, with novel variants displaying substantial antigenic differences, further studies should be aimed to monitor the performances of the serological tests for the ability to correctly assess the serological status of susceptible animal populations.

## Figures and Tables

**Table 1:** Sampling details.

Sampling reference	Days of collection	Number of sera collected
A	0	14
B	7	19
C	65	22
D	307	29

**Table 2:** Details of serological methods.

No.	Name	Target protein	References
1	Plaque reduction neutralisation test (PRNT)	S/N	[21]
2	SARS-CoV-2 Spike LIPS (LIPS-S)	S	[7]
3	SARS-CoV-2 Nucleocapsid LIPS (LIPS-N)	N	[7]
4	ID screen® SARS-CoV-2 Double Antigen Multi-Species ELISA (IDV)	N	ID.vet, https://www.id-vet.com
5	ERADIKIT™ COVID19-MULTISPECIES (Eradikit)	N	In3Diagnostic, https://www.in3diagnostic.com

**Table 3:** Cohen's kappa agreements between LIPS-S, LIPS-N, PRNT, IDV, and Eradikit.

Cohen's kappa					
Methods	LIPS-S	LIPS-N	PRNT	IDV	Eradikit
LIPS-S	1	0.2845	1	0.08501	0.162
LIPS-N	0.2845	1	0.2845	0.4267	0.56
PRNT	1	0.189	1	0.08501	0.162
IDV	0.08501	0.4267	0.08501	1	0.414
Eradikit	0.162	0.56	0.162	0.414	1

## Data Availability

Data can be made available upon request to the corresponding author.

## References

[1] Oude Munnink B. B., Sikkema R. S., Nieuwenhuijse D. F. (2021). Transmission of SARS-CoV 2 on mink farms between humans and mink and back to humans. *Science*.

[2] EFSA (2021). *European Food Safety Authority and European Centre for Disease Prevention and Control*.

[3] FAO (2022). *Animal Production and Health, SARS-CoV-2 in Animals*.

[4] Michelitsch A., Wernike K., Ulrich L., Mettenleiter T. C., Beer M. (2021). SARS-CoV 2 in animals: from potential hosts to animal models. *Advances in Virus Research*.

[5] Padilla-Blanco M., Aguiló-Gisbert J., Rubio V. (2022). The finding of the severe acute respiratory syndrome coronavirus (SARS-CoV 2) in a wild eurasian river otter (Lutra lutra) highlights the need for viral surveillance in wild mustelids. *Frontiers in Veterinary Science*.

[6] WOAH (2022). Considerations on monitoring SARS-CoV 2 in animals. https://en-sars-cov-2-surveillance.pdf.

[7] Berguido F. J., Burbelo P. D., Bortolami A. (2021). Serological detection of SARS-CoV 2 antibodies in naturally-infected mink and other experimentally-infected animals. *Viruses*.

[8] Meyer B., Drosten C., Müller M. A. (2014). Serological assays for emerging coronaviruses: challenges and pitfalls. *Virus Research*.

[9] Geurtsvan-Kessel C. H., Okba N. M. A., Igloi Z. (2020). An evaluation of COVID-19 serological assays informs future diagnostics and exposure assessment. *Nature Communications*.

[10] Taylor S. C., Hurst B., Charlton C. L. (2021). A new SARS-CoV 2 dual-purpose serology test: highly accurate infection tracing and neutralizing antibody response detection. *Journal of Clinical Microbiology*.

[11] Embregts C. W. E., Verstrepen B., Langermans J. A. M. (2021). Evaluation of a multi-species SARS-CoV 2 surrogate virus neutralization test. *One Health*.

[12] Quinti I., Mortari E. P., Fernandez Salinas A., Milito C., Carsetti R. (2021). IgA antibodies and IgA deficiency in SARS-CoV-2 infection. *Frontiers in Cellular and Infection Microbiology*.

[13] Pulido J., García-Durán M., Fernández-Antonio R. (2022). Receptor-binding domain-based immunoassays for serosurveillance differentiate efficiently between SARS-CoV 2-exposed and non-exposed farmed mink. *Journal of Veterinary Diagnostic Investigation*.

[14] Perkmann T., Koller T., Perkmann-Nagele N. (2021). Spike protein antibodies mediate the apparent correlation between SARS-CoV 2 nucleocapsid antibodies and neutralization test results. *Microbiology Spectrum*.

[15] Badiola J. J., Otero A., Sevilla E. (2021). SARS-CoV 2 outbreak on a Spanish mink farm: epidemiological, molecular, and pathological studies. *Frontiers in Veterinary Science*.

[16] Cardillo L., de Martinis C., Brandi S. (2022). SARS-CoV 2 serological and biomolecular analyses among companion animals in campania region (2020–2021). *Microorganisms*.

[17] Decaro N., Grassi A., Lorusso E. (2021). Long-term persistence of neutralizing SARS-CoV 2 antibodies in pets. *Transboundary and Emerging Diseases*.

[18] Volkova M. A., Zinyakov N. G., Yaroslavtseva P. S., Chvala I. A., Galkina T. S., Andreychuk D. B. (2021). Development of the test kit for detection of SARS-CoV 2 antibodies in sera of susceptible animals. *Veterinary Science Today*.

[19] Fenollar F., Mediannikov O., Maurin M. (2021). Mink, SARS-CoV 2, and the human-animal interface. *Frontiers in Microbiology*.

[20] Hammer A. S., Quaade M. L., Rasmussen T. B., Fonager J., Rasmussen M., Mundbjerg K. (2021). SARS-CoV 2 transmission between mink (neovison vison) and humans, Denmark. *Emerging Infectious Diseases*.

[21] Padoan A., Bonfante F., Cosma C. (2021). Analytical and clinical performances of a SARS-CoV 2 S-RBD IgG assay: comparison with neutralization titers. *Clinical Chemistry and Laboratory Medicine*.

[22] Corman V. M., Landt O., Kaiser M. (2020). Detection of 2019 novel coronavirus (2019-NCoV) by real-time RT-PCR. *Euro Surveillance*.

[23] Burbelo P. D., Riedo F. X., Morishima C. (2020). Sensitivity in detection of antibodies to nucleocapsid and spike proteins of severe acute respiratory syndrome coronavirus 2 in patients with coronavirus disease 2019. *The Journal of Infectious Diseases*.

[24] Lex A., Gehlenborg N., Strobelt H., Vuillemot R., Pfister H. (2014). UpSet: visualization of intersecting sets. *IEEE Transactions on Visualization and Computer Graphics*.

[25] Conway J. R., Lex A., Gehlenborg N. (2017). UpSetR: an *R* package for the visualization of intersecting sets and their properties. *Bioinformatics*.

[26] WOAH (2020). Infection with SARS-CoV 2 in animals. https://EN_Factsheet_SARS-CoV-2.pdf.

[27] Zhao S., Schuurman N., Li W. (2021). Serologic screening of severe acute respiratory syndrome coronavirus 2 infection in cats and dogs during first coronavirus disease wave, Netherlands. *Emerging Infectious Diseases*.

[28] Colitti B., Bonfante F., Grazioli S. (2022). Detailed epitope mapping of SARS-CoV 2 nucleoprotein reveals specific immunoresponse in cats and dogs housed with COVID-19 patients. *Research in Veterinary Science*.

[29] Giner J., Villanueva-Saz S., Tobajas A. P. (2021). SARS-CoV 2 seroprevalence in household domestic ferrets (*Mustela putorius* furo). *Animals*.

[30] Hamer S. A., Nunez C., Roundy C. M. (2022). Persistence of SARS-CoV 2 neutralizing antibodies longer than 13 months in naturally infected, captive white-tailed deer (*Odocoileus virginianus*), Texas. *Emerging Microbes and Infections*.

[31] Colitti B., Bertolotti L., Mannelli A. (2021). Cross-sectional serosurvey of companion animals housed with SARS-CoV 2–infected owners, Italy. *Emerging Infectious Diseases*.

[32] Harrington W. E., Trakhimets O., Andrade D. V. (2021). Rapid decline of neutralizing antibodies is associated with decay of IgM in adults recovered from mild COVID-19. *Cell Reports Medicine*.

[33] Wheatley A. K., Juno J. A., Wang J. J. (2021). Evolution of immune responses to SARS-CoV 2 in mild-moderate COVID-19. *Nature Communications*.

[34] Galkina T. S., Nesterov A. A., Borisov A. V., Chvala I. A., Kononov A. V. (2021). Development of carnivac-Cov vaccine against coronavirus infection (COVID-19) in carnivores. *Veterinary Science Today*.

